# Isolated Asymptomatic Metastatic Melanoma to the Colon: A Case Report

**DOI:** 10.7759/cureus.4109

**Published:** 2019-02-20

**Authors:** Passisd Laoveeravat, Nicha Wongjarupong, Lisa Smith, Mitchell S Wachtel, Sameer Islam

**Affiliations:** 1 Internal Medicine, Texas Tech University Health Sciences Center, Lubbock, USA; 2 Internal Medicine, University of Minnesota, Minneapolis, USA; 3 Pathology, Texas Tech University Health Sciences Center, Lubbock, USA; 4 Gastroenterology, Texas Tech University Health Sciences Center, Lubbock, USA

**Keywords:** metastatic melanoma, colon, isolated

## Abstract

Metastatic melanoma is generally rare, and the colon is a very rare metastatic site. We report a case of asymptomatic, isolated metastatic melanoma to the colon. Asymptomatic patients are usually not expected to have metastatic lesions in the colon. Ninety-five percent of large bowel metastases are identified during a postmortem examination. Our patient was found to have metastatic melanoma to the colon during a follow-up colonoscopy done for the surveillance of colon polyps. An awareness that patients with melanoma may possibly develop colon metastases is needed.

## Introduction

Metastatic melanoma to the colon is rare. The leading cause of metastatic cancer to the colon is breast cancer, and melanoma itself often spreads to the small bowel rather than to the colon [1]. The presentation of patients includes bowel obstruction, perforation, lower gastrointestinal bleeding, and constitutional symptoms. Most of the patients with metastatic melanoma to the colon were diagnosed by a postmortem finding [1-2].

We present a case of a middle-aged male who was asymptomatic and found to have a colon mass on surveillance colonoscopy with a diagnosis of metastatic melanoma on histopathology.

## Case presentation

A 59-year-old Caucasian male with a past medical history of auricular malignant melanoma underwent excision of the lesion and sentinel lymph node biopsy at the age of 52. He had lymph node metastasis and underwent neck lymph nodes dissection. At the age of 55, he underwent screening colonoscopy, which showed two polyps in the descending and sigmoid colon. The pathology revealed an adenomatous polyp without dysplasia. He was asymptomatic without anemia, change in bowel habit, or weight loss at the time of the screening colonoscopy. His brother had a history of colon cancer diagnosed at age 62. He does not have Ashkenazi Jewish ancestry. Poster presentation: Laoveeravat P, Wongjarupong N, Suchartlikitwong S, Mingbunjerdsuk T, Vutthikraivit W, El Nawaa S, Smith L, Wachtel M, Islam S. Isolated asymptomatic metastatic melanoma to the colon: a case report. ACG Conference; October 9, 2018.

**Figure 1:**
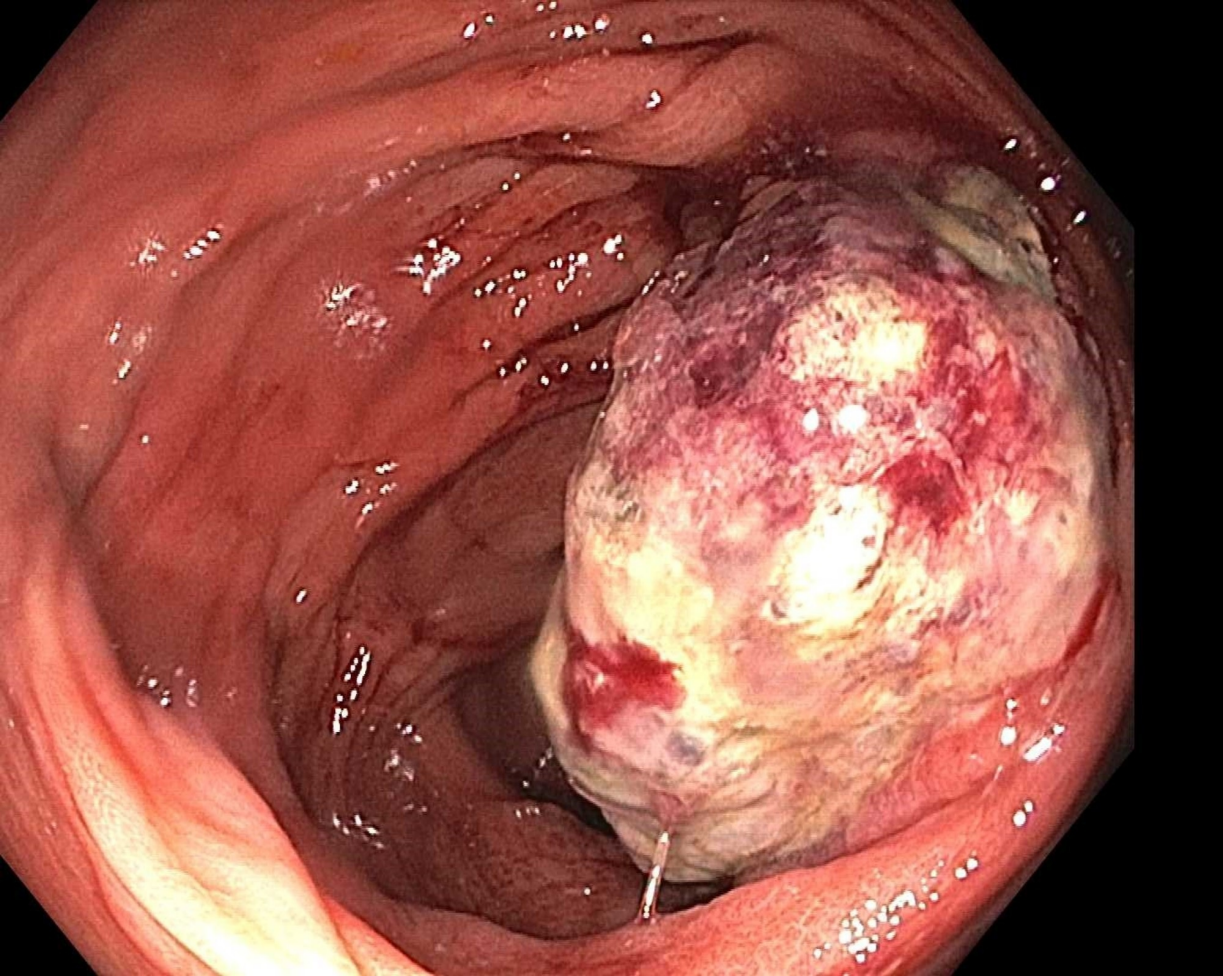
A polypoid and ulcerated non-obstructing mass at the hepatic flexure.

**Figure 2:**
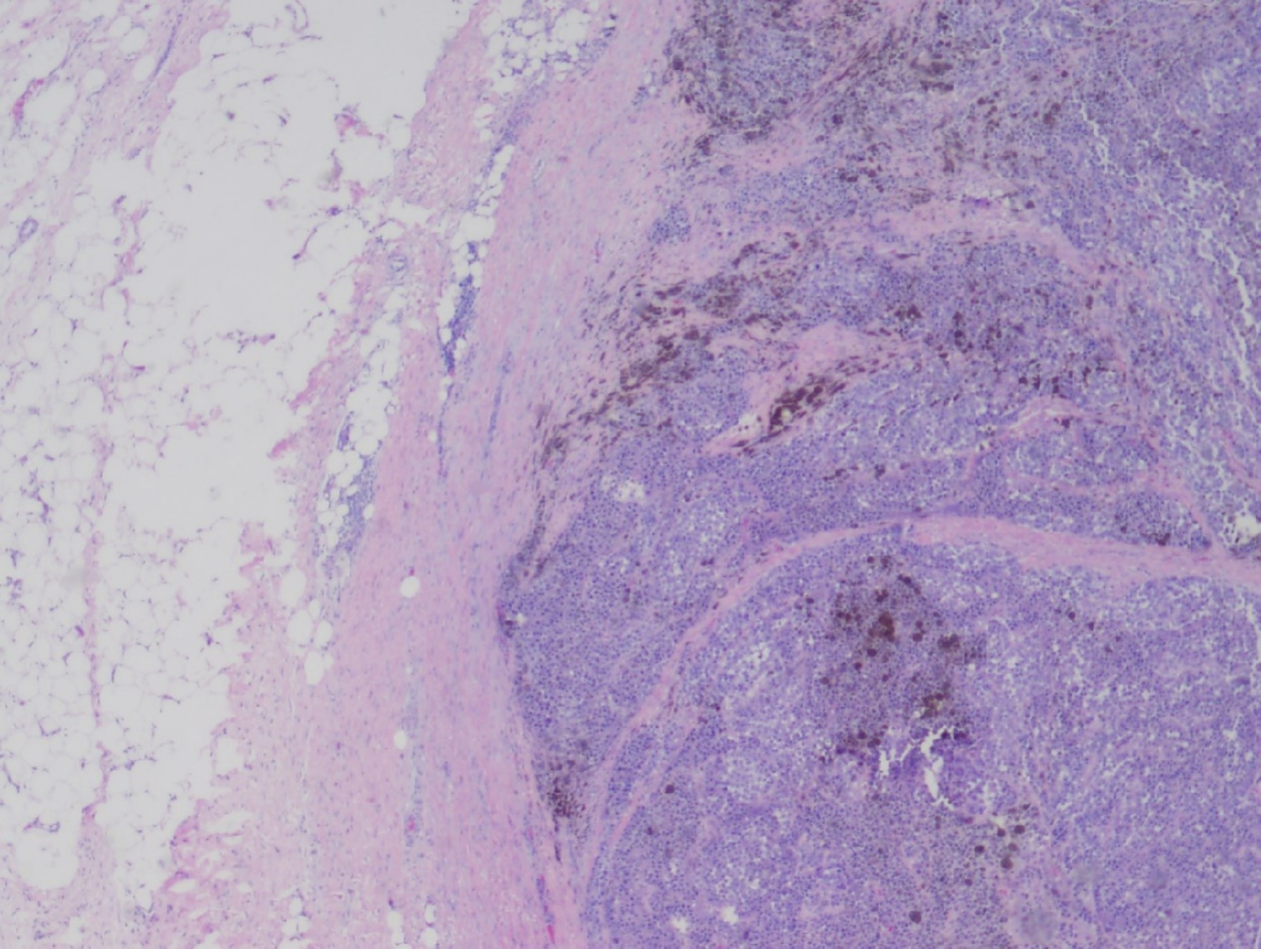
A predominantly mucosal/ submucosal mass with brown pigments (H&E, 25X).

**Figure 3:**
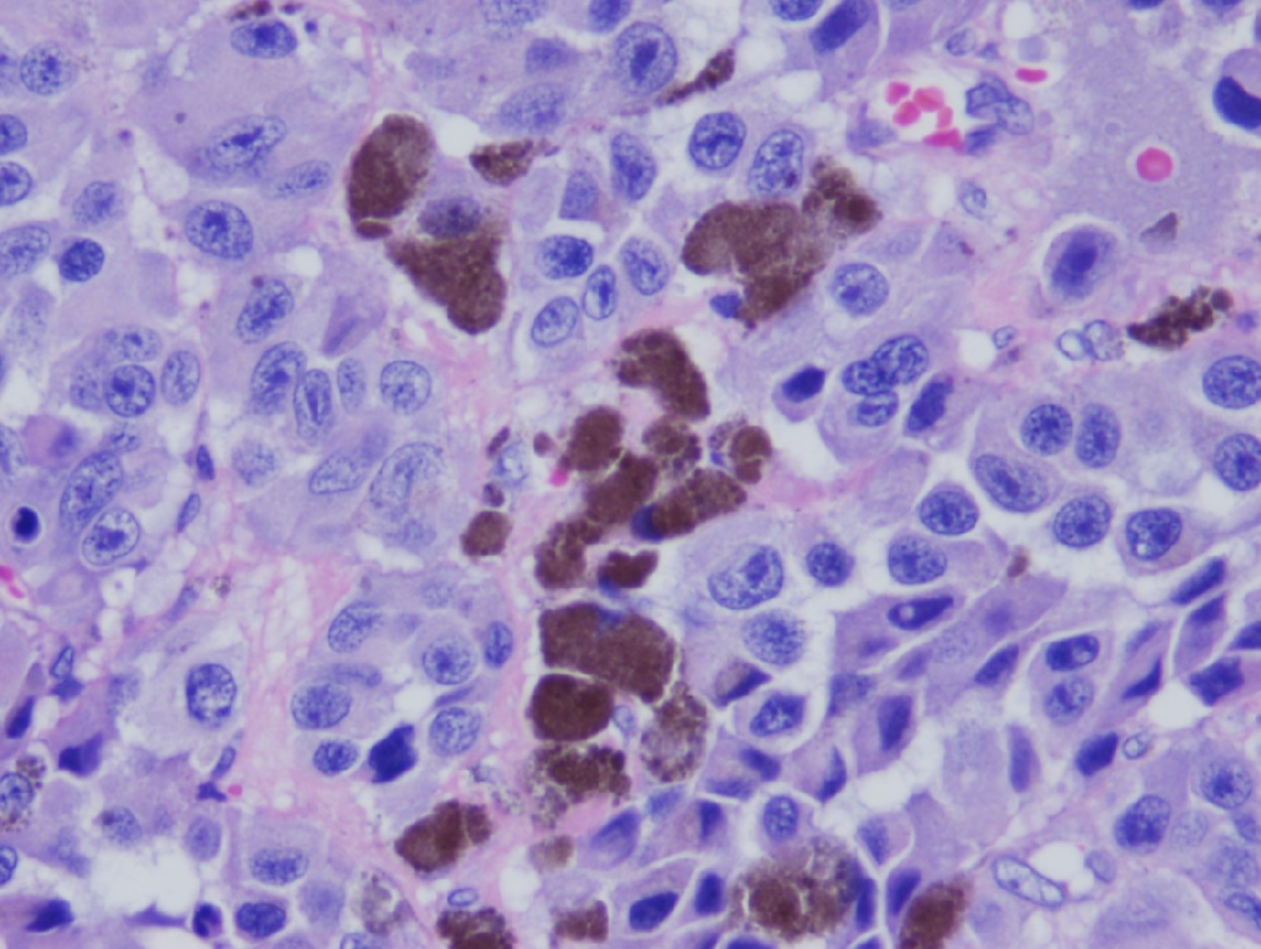
The brown pigments lie almost entirely in macrophages. Anaplastic cancer cells showed large, round to ovoid nuclei with large nucleoli, sometimes multiple, often eccentrically placed in amphophilic cytoplasm (H&E, 400X).

**Figure 4:**
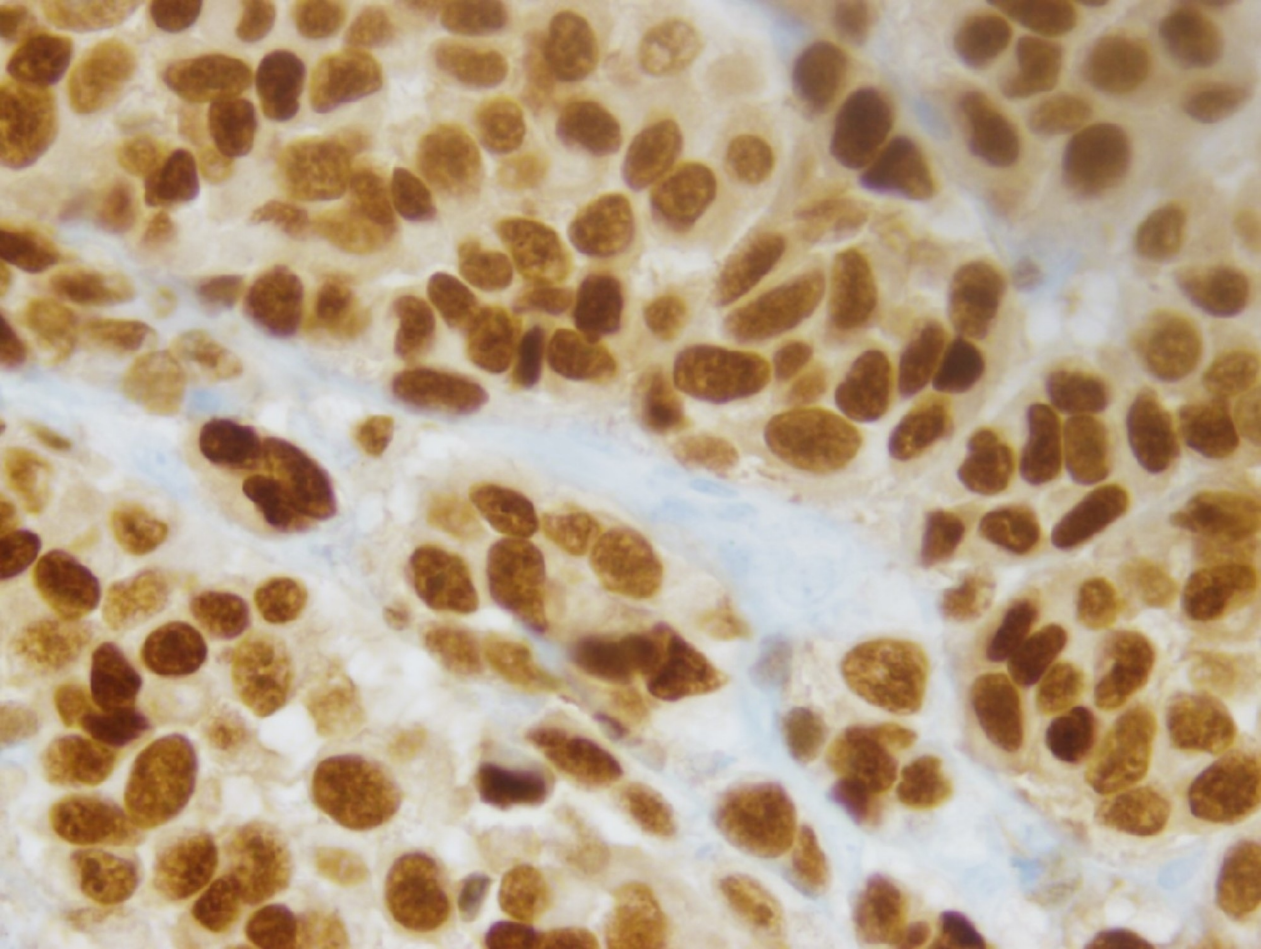
Cancer cell nuclei uniformly expressed SOX10 (immunohistochemical stain, 400X). SOX10; SRY Box 10

Two years later, he returned for a follow-up colonoscopy at the age of 57 according to the gastroenterologist's preference. He remained asymptomatic. Laboratory results revealed a normal hemoglobin level of 13.7 g/dL. He was found to have 4x4x2 cm frond-like, polypoid, and ulcerated non-obstructing mass at the hepatic flexure (Figure 1). The histological results showed suspected melanoma. He also underwent gastroscopy, which did not show any abnormalities. He underwent a laparoscopic right hemicolectomy and omentectomy. Histologic examination revealed a predominantly mucosal/submucosal mass with brown pigments (Figure 2). The brown pigments lay almost entirely in the macrophages. Anaplastic cancer cells showed large, round to ovoid nuclei with large nucleoli, sometimes multiple, often eccentrically placed in amphophilic cytoplasm (Figure 3), and cancer cell nucleoli uniformly expressed SRY-Box 10 (SOX10) (Figure 4). The SOX10 stain was positive, which was consistent with a diagnosis of melanoma. Moreover, two out of 18 intra-abdominal lymph nodes were positive for malignant melanoma. The immuno-histochemistry and molecular genetics tests were negative for neuroblastoma RAS (NRAS), cytokeratin 7 (CK7), cytokeratin 20 (CK20), p63, chromogranin, thyroid transcription factor-1 (TTF-1), and synaptophysin and positive for c-kit.

Positron emission tomography/computed tomography scan (PET/CT) one month prior to the last colonoscopy did not find areas of increased fluoro-deoxyglucose (FDG) uptake suspected of metastasis but a focal area of increased FDG uptake at the splenic flexure indicating diverticulitis. CT abdomen four months prior to the colonoscopy was normal. The patient was treated with a combination of immunotherapy, including nivolumab and ipilimumab.

## Discussion

Colonic involvement of metastatic melanoma is rare, with an incidence of 0.18%-2.1% [3-6], and it is the least common site of gastrointestinal (GI) tract metastasis for this cancer [1-2,6-7]. The locations commonly involved with melanoma in the GI tract are the small bowel and rectum [1-2,7].

Our patient was asymptomatic, and we found metastatic melanoma during a surveillance colonoscopy without prior suspicion. The mean age of the large bowel metastasis diagnosis ranges from 56 to 60 years. Eighty percent of patients were diagnosed with a symptomatic metastatic lesion in the colon before the age of 50. There are various presentations of metastatic melanoma to the gastrointestinal tract. Fifty-one percent of symptomatic patients present with lower gastrointestinal bleeding [6]. Other, less common symptoms include anemia without obvious bleeding, weight loss, and bowel obstruction / perforation [6]. Many patients remain undiagnosed until the autopsy [1,7]. Fifteen percent of patients diagnosed premortem are asymptomatic [4]. The survival rate seems to depend on the presenting symptoms, with poor survival among patients who presented with bowel obstruction or perforation, with an average life expectancy of 10 months [6]. However, there were no data regarding asymptomatic patients, as most were diagnosed postmortem, during the autopsy.

In our case, the patient initially presented with a colon polyp without malignant histology. He developed a mass at the hepatic flexure two years after the first colonoscopy. The time from melanoma diagnosis to metastatic disease was five years in our patient. The average time was 7.47 years (range 1-41 years) and 12.5% of the patients developed metastatic melanoma solely to the large bowel [6]. However, Park et al. reported that the large bowel as the single site of metastasis was found in 47% of patients [4].

Endoscopic findings of metastatic melanoma vary from mass to polyp [1,6]. Our patient was found to have a mass at the hepatic flexure. There are no previous reports describing the hepatic flexure as a metastatic colon site. However, the ascending colon and the transverse colon have been reported as metastatic sites.

A previous report showed the sensitivity and specificity of a PET/CT scan for the detection of metastatic melanoma were 91% and 92%, respectively [8]. However, in our case, the prior PET/CT scan was normal and did not suggest a metastatic lesion. More aggressive endoscopic workup should be considered in patients with a history of metastatic melanoma who present with any alarming lower gastrointestinal symptoms, such as anemia, bleeding per rectum, changes in bowel habits, or weight loss.

## Conclusions

Metastatic melanoma to the colon is rarely found during a premortem examination. The interval from the diagnosis of melanoma to finding a metastatic disease in the colon is usually long. Patients with metastatic melanoma should be carefully screened for gastrointestinal symptoms and a timely colonoscopy may benefit these patients in detecting early metastatic disease.
